# Impact of Interspecific Hybridization between Crops and Weedy Relatives on the Evolution of Flowering Time in Weedy Phenotypes

**DOI:** 10.1371/journal.pone.0014649

**Published:** 2011-02-03

**Authors:** Corinne Vacher, Tanya M. Kossler, Michael E. Hochberg, Arthur E. Weis

**Affiliations:** 1 INRA, UMR1202 Biodiversité Gènes et Communautés, Cestas, France; 2 Université Montpellier II, UMR5554 Institut des Sciences de l'Evolution, Montpellier, France; 3 Department of Ecology and Evolutionary Biology, University of California Irvine, Irvine, California, United States of America; 4 Department of Biology, Duke University, Durham, North Carolina, United States of America; 5 Department of Ecology and Evolutionary Biology, University of Toronto, Toronto, Canada; University Copenhagen, Denmark

## Abstract

**Background:**

Like conventional crops, some GM cultivars may readily hybridize with their wild or weedy relatives. The progressive introgression of transgenes into wild or weedy populations thus appears inevitable, and we are now faced with the challenge of determining the possible evolutionary effects of these transgenes. The aim of this study was to gain insight into the impact of interspecific hybridization between transgenic plants and weedy relatives on the evolution of the weedy phenotype.

**Methodology/Principal Findings:**

Experimental populations of weedy birdseed rape (*Brassica rapa*) and transgenic rapeseed (*B. napus*) were grown under glasshouse conditions. Hybridization opportunities with transgenic plants and phenotypic traits (including phenological, morphological and reproductive traits) were measured for each weedy individual. We show that weedy individuals that flowered later and for longer periods were more likely to receive transgenic pollen from crops and weed×crop hybrids. Because stem diameter is correlated with flowering time, plants with wider stems were also more likely to be pollinated by transgenic plants. We also show that the weedy plants with the highest probability of hybridization had the lowest fecundity.

**Conclusion/Significance:**

Our results suggest that weeds flowering late and for long periods are less fit because they have a higher probability of hybridizing with crops or weed×crop hybrids. This may result in counter-selection against this subset of weed phenotypes, and a shorter earlier flowering period. It is noteworthy that this potential evolution in flowering time does not depend on the presence of the transgene in the crop. Evolution in flowering time may even be counter-balanced by positive selection acting on the transgene if the latter was positively associated with maternal genes promoting late flowering and long flowering periods. Unfortunately, we could not verify this association in the present experiment.

## Introduction

When transgenic plants were initially developed, most plant evolutionary biologists and geneticists considered spontaneous hybridization between species to be rare and of little importance in terms of evolution. This view extended to both crops and their wild or weedy relatives [1], but has now radically changed. More than twenty years of gene-flow research has shown that interspecific hybridization is very common in some groups of vascular plants [2], [3] and may be of considerable evolutionary significance. Hybridization may occasionally result in the extinction of a population [1], [4], may trigger the evolution of plant invasiveness [5], or initiate speciation [6], [7]. A substantial body of evidence [8], [9] has now accumulated, demonstrating the high potential for interspecific hybridization between agricultural crops and their wild or weedy relatives. Transgenic crops are no exception, and empirical studies have provided evidence of transgene dispersal from GM crops to their weedy relatives [10], [11], [12], [13], [14].

Many factors have been shown to influence the rate of hybrid formation between crops and their wild or weedy relatives. Population effects such as the local densities of the parental types and their relative frequencies, have been demonstrated in several cases [12], [15], [16], [17], [18], [19]. Mating system differences at the individual level due to, for example, selfing rates and apomixis, have also been found to affect hybridization rates [20]. Moreover, several studies have shown that overlap in the flowering periods of crop and weed plants affect opportunities for hybridization [18], [21].

The aim of this study is to gain insight into the impact of hybridization with transgenic crops on the evolution of the weedy relatives by (1) verifying that hybridization opportunities for weedy plants depend on their phenotypic traits (including flowering phenology), (2) measuring the relative fitness of hybridizing weeds, and (3) searching for associations between the transgenic trait and the phenotypic traits increasing hybridization opportunities in the offspring of weedy plants.

We studied hybridization opportunities, phenotypic traits (including phenological, morphological and reproductive traits) and offspring phenotype of weedy individuals (Table 1) in experimental plant populations cultivated under glasshouse conditions. Experimental populations were composed of weeds (birdseed rape, *Brassica rapa* L., AA, 2n = 20) and transgenic plants in a 1∶1 ratio. Transgenic plants were crop plants of the *Brassica* genus (rapeseed, *Brassica napus* L. *ssp oleifera*, AACC, 2n = 38), F1 hybrids between *B. rapa* and *B. napus*, or first-generation backcrosses. Crop plants were all homozygous for the *Btcry1Ac* transgene from *Bacillus thuringiensis* (*Bt*) [22], F1 hybrids were all hemizygous and first-generation backcrosses and consisted of an equal mixture of hemizygotes and null homozygotes. Hybridization opportunities for each weedy individual was calculated as the expected proportion of pollen received from transgenic plants (*PPR*) based on the observed flowering schedules.

**Table 1:** Phenotypic traits studied in weedy mother plants (M) and their offspring (O).

Trait	Generation
Time to first flower	M, O
Flowering duration	M
Stem diameter on the day of first flower	M, O
Stem height on the day of first flower	M
Total number of filled seeds	M
Expression of the *Bt-*transgene	O

This experimental system was ideal for addressing the question of interest in this study, for three reasons. First, despite barriers to interspecific mating such as apomixis [20] or preferential exclusion of hybrid zygotes [23], numerous studies [24] have shown that *B. napus* and *B. rapa* readily hybridize under controlled conditions, but also in the field. Spontaneous hybridization has, for instance, been reported in weedy populations of *B. rapa* growing in agricultural crops [12], [25], [26] and in natural populations of *B. rapa* occurring near waterways [27]. Second, flowering time has been extensively studied in *B. rapa* [18], [28], [29], and temporal clines in phenotypic traits have been observed. For example, time to first flowering has been shown to be positively correlated with stem height and stem diameter [28], [30], [31]. Third, transgenic lines of *B. napus* containing a green fluorescent protein (GFP) gene associated with the *Bt* transgene have been constructed [32], [33]. The presence of the *Bt* transgene in the offspring of weedy plants can therefore be inferred by exposing the plants to UV light [32], [34].

## Results

### (1) Relationship between hybridization opportunities for weedy individuals, their flowering phenology, and their morphology

**Figure 1:**
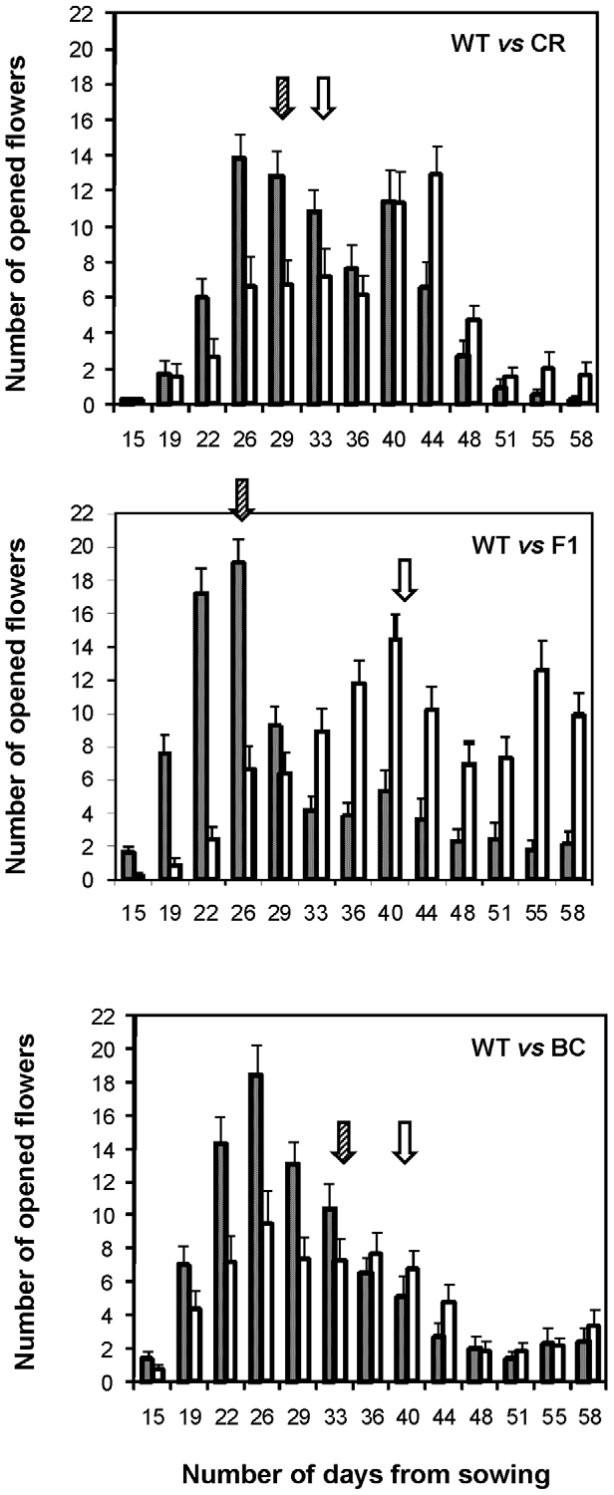
Phenology of transgenic and weedy plants. Phenology of weedy plants (WT; *hatched bars*) and their *Bt*-transgenic relatives (CR, F1 or BC; *white bars*). For each combination, three mixed populations of 30 plants were monitored. Bars represent are the mean numbers of opened flowers per population for each day of observation, with standard errors. WT: weedy plants of *B. rapa*; CR: *Bt*-crop plants of *B. napus*; F1: F1 hybrids between WT and CR; BC: *Bt*-plants from the backcross of F1 on WT. Arrows indicate the date at which 50% of the flowers had been produced.

As expected from previous results [18], weeds flowered earlier than transgenic crops and hybrids (Fig. 1), with the F1 hybrids flowering the latest. Correspondingly, the expected proportion of crosses between weeds and F1 plants was lower than that for crop or backcross plants (Table 2A). Moreover, *PPR* (log transformed) increased with the time to first flower and the duration of flowering in weedy individuals (see overall slopes in Table 2B). The overall slope for the interaction between the two phenological traits (Table 2B) was close to zero and did not qualitatively modify these effects. However, significant interactions (Table 3) indicated that the effects of phenology of weedy plants on *PPR* depended on transgenic type (crop, F1 hybrid or first-generation backcross). The regression coefficients and their 95% confidence limits indicated that a longer time to flowering and a longer flowering duration increased *PPR* more for F1 hybrids than for crops or first-generation backcrosses (see within-type slopes in Table 2B). Thus, weedy individuals flowering later and for longer periods were more likely to receive transgenic pollen, particularly if the transgenic donors were first-generation crop x weed F1 hybrids.

**Table 2:** Effects of phenological traits on the expected proportion of pollen received from transgenic plants (*PPR*) of weedy *B. rapa* in mixed populations including transgenic *B. napus* crop and crop-weed hybrids.

A. Means	Overall	Transgenic type		
		Crop	F1	Backcross
Expected proportion of pollinations by transgenic plants (*PPR*)	0.39	0.39	0.36	0.41
± Standard error	±0.01	±0.01	±0.02	±0.02
B. Slopes				
Time to first flower	**0.06**	0.01	**0.21**	0.11
95% C.L.	(0.03; 0.09)	(−0.05; 0.06)	(0.06; 0.36)	(−0.03; 0.25)
Flowering duration	**0.16**	0.02	**0.46**	0.24
95% C.L.	(0.07; 0.26)	(−0.15; 0.20)	(0.02; 0.90)	(−0.18; 0.65)
Time x duration	**−0.005**	0.00	**−0.02**	−0.01
95% C.L.	(−0.01; 0.00)	(−0.01; 0.01)	(−0.04; 0.00)	(−0.03; 0.01)

**A.** The mean *PPR* for the transgenic type treatments. **B.** The influence of weedy traits. “Slopes” are the coefficients for the effect of each trait on *PPR*. The “overall” slope indicates the effect across all transgenic types. The within-type slopes were obtained from the mixed linear model presented in Table 3, they indicate the relationships for crop, F1 and backcross migrants. Coefficients that do not include zero in their 95% confidence interval are shown in bold typeface.

**Table 3:** Mixed linear model for the effects of transgenic type and weedy plant phenological traits on the expected proportion of pollen received from transgenic plants (*PPR*).

Source	df (numerator)	df (denominator)	F value	P
Transgenic type	2	105	8.28	0.0005
Time to first flower	1	99.2	21.25	<.0001
Flowering duration	1	99.5	27.63	<.0001
Type × time	2	99.2	5.41	0.0059
Type × duration	2	99.4	8.52	0.0004
Time × duration	1	99.3	14.07	0.0003
Type × time × duration	2	99.3	5.47	0.0056

−2 residual log likelihood  = −35.1.

Akaike's information criterion  = −31.1.

As expected from the results of previous studies [28], [30], we observed temporal clines in the morphological traits under study. Time to first flower was positively correlated with stem diameter (*r*_s_ = 0.31, P<0.001) and stem height (*r*_s_ = 0.18, P<0.05). These correlations indicate that the opportunity for hybridization may not be random, and may instead depend on the morphology of the weed. We found a significant, single effect of stem diameter on *PPR* (F_1,105_ = 5.0, P<0.05). The overall slope was positive and its 95% confidence interval did not include zero (slope = 0.05, CL = (0.01, 0.09)), indicating that plants with large stems on the day of the first flower were more likely to hybridize with transgenic plants. No such effect was detected for stem height, either as a single effect (F_1,104_ = 0.05, P = 0.82) or in interaction with transgenic type (F_2,104_ = 0.87, P = 0.42).

### (2) Relative fitness of hybridizing weeds

**Figure 2:**
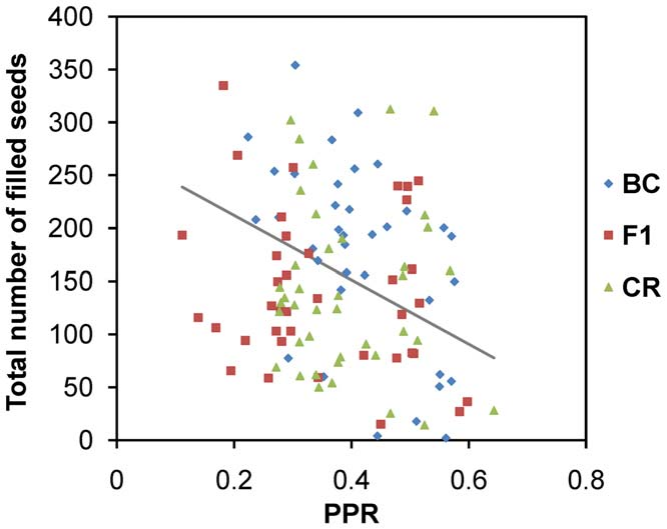
Decrease in fecundity with *PPR*. Total number of filled seeds (*TNS*) produced by weedy individuals as a function of the expected proportion of pollen (*PPR*) received from transgenic plants (CR: *Bt*-crop plants of *B. napus*; F1: F1 hybrids between weedy plants and CR; BC: *Bt*-plants from the backcross of F1 on weedy plants). The grey line corresponds to the regression line across all transgenic types (*TNS*  = −303.15 *PPR* +272.69); its slope is the overall slope given in Table 5.

For any given weedy plant in the experimental populations, the total number of filled seeds decreased significantly with *PPR* (see overall slopes in Table 4B and the significant effect of *PPR* in Table 5). We observed no significant interaction between *PPR* and transgenic type (Table 5), indicating that this decrease in fecundity with *PPR* was not dependant on transgenic type (crop, F1 hybrid or first-generation backcross). This decrease in fecundity was observed despite the positive correlation between *PPR* and total flower production within weedy plants (*r*_s_ = 0.37, *P*<0.001). An alternative analysis (not shown), including transgenic type as fixed effect and phenological traits of weeds (time to first flower or flowering duration) as covariates also predicted the total number of filled seeds. We found a significant effect of the time to first flower on the number of seeds, in interaction with transgenic type (F_2,107_ = 3.66, P<0.05). However, all the 95% confidence intervals of the regression coefficients for each transgenic type included zero, making further interpretation impossible. Flowering duration was significant as a single effect (F_1,105_ = 37.4, P<0.001). The overall slope was negative and its 95% confidence interval did not include zero (slope = −21.3, CL = (−28.77, −14.51)), indicating that weedy plants with longer flowering times produced fewer seeds. Thus, the weedy plants with the highest probability of being pollinated by *Bt-*transgenic plants were those with the lowest fecundity (Fig. 2).

**Table 4:** Effect of the expected proportion of pollen received from transgenic plants (*PPR*) on the total number of filled seeds produced by weedy *B. rapa* in mixed populations including transgenic *B. napus* crop and crop-weed hybrids.

A. Means	Overall	Transgenic type		
		Crop	F1	Backcross
Total number of seeds	151.68	140.23	140.26	176.44
± standard error	±7.63	±12.58	±12.00	±14.60
B. Slopes				
*PPR*	**−303.15**	**−372.1**	−319.21	−290.42
95% C.L.	(−466.73; −139.57)	(−729.13; −15.07)	(−1098.72; 460.30)	(−1160.18; 579.34)

**A.** Mean seed production for the transgenic type treatments. **B.** The influence of *PPR*. “Slopes” are the coefficients for the effect of each trait on seed production. The “overall” slope indicates the effect across all transgenic types. The within-type slopes were obtained from the mixed linera model presented in Table 5, they indicate the relationships for crop, F1 and backcross plants. Coefficients that do not include zero in their 95% confidence interval are in shown in bold typeface.

**Table 5:** Mixed linear model for the effects of transgenic type and expected proportion of pollen received from transgenic plants (*PPR*) on the total number of filled seeds.

Source	df (numerator)	df (denominator)	F value	P
Transgenic type	2	28.7	0.07	0.929
*PPR*	1	74.3	12.19	0.0008
Transgenic type x *PPR*	2	76.2	0.05	0.948

−2 residual log likelihood  = 1240.8.

Akaike's information criterion  = 1244.8.

### (3) Associations between the transgenic trait and the phenotypic traits increasing hybridization opportunities in the offspring of weedy individuals

An analysis of offspring phenotype showed that time to first flower in weedy mother plants had a significant effect on the average time to first flower of their offspring (F_1,104_ = 7.48, P<0.05). Transgenic type (crop, F1 hybrid or first-generation backcross) did not affect time to first flowering in the offspring, either as a main effect (F_2,71.3_ = 0.20, P = 0.82) or in interaction with maternal time to first flower (F_2,97.4_ = 0.40, P = 0.66). In contrast, offspring stem diameter was not affected by maternal diameter (F_1,104_ = 1.80, P = 0.18) or maternal time to first flower (F_1,104_ = 2.03, P = 0.15). These results confirm that late-flowering plants tend to produce late-flowering offspring [28]. Because late-flowering plants were also more likely to receive transgenic pollen, we therefore expected to find more transgenic offspring in the offspring of late-flowering weedy mothers and an association between the transgenic trait and time to first flower in the offspring generation.

Contrary to expectation, we found no evidence to suggest that weedy individuals with higher *PPR* produced more transgenic offspring. A total of 1648 seedlings, obtained from 126 weedy plants, were scored under UV light for the *Bt*-GFP construct. Only 38 seedlings, produced by 17 weedy mothers, scored positively. None of them was sired by the pollen of F1 hybrids. The proportions of fluorescent seedlings were equal to 0.04±0.01 for populations with crop plants, 0.0±0.0 for populations with F1 hybrids and 0.02±0.01 for populations with first-generation backcrosses (χ^2^ = 17.55, df = 2, P<0.001). Significant differences were observed between replicates for the proportion of positive scores (χ^2^ = 15.76, df = 2, P<0.001). The proportion of *Bt*-GFP+ seedlings was not correlated with *PPR* in populations with crop plants (*r*_s_ = −0.16, P = 0.33) nor with backcross plants (*r*_s_ = 0.23, P = 0.17). Correlations with the proportion of *Bt*-GFP+ seedlings were also weak and non significant for all other maternal traits measured. Thus, variation in the probability of weedy mother plants being pollinated by transgenic donors did not translate into variation in the proportion of *Bt*-seedlings in their offspring.

Because of the very low proportions of *Bt-*GFP+ seedlings, we could not study the associations between the transgenic trait and the phenotypic traits increasing hybridization opportunities in the offspring of weedy plants. Among the 1654 seedlings scored under UV light, 1048 reached the first flower stage and were measured. Unfortunately, only nine of these plants were *Bt*-GFP+, and seven of these nine plants were half sibs (the nine plants were produced by only three weedy mothers). The 31 remaining *Bt*-GFP+ seedlings did not reach the first flower stage. There were, therefore, clearly too few *Bt*-GFP+ plants to compare the phenotypic characteristics of *Bt-*GFP+ and *Bt-*GFP- offspring.

## Discussion

The aim of our experiment was to assess the impact of interspecific hybridization between weedy *B. rapa* and transgenic *B. napus* on the evolution of the weedy phenotype. This was done by identifying the phenotypic traits increasing hybridization opportunities for weedy individuals, searching for associations between thesephenotypic traits and the transgenic trait in the offspring of weedy mothers and evaluating the relative fitness of hybridizing weeds. Our results show that weedy individuals that flowered later and for longer periods were more likely to receive transgenic pollen from crops and weed×crop hybrids. Because stem diameter is correlated with flowering time [28], [30], plants with wider stems were also more likely to be pollinated by transgenic plants. Our results suggest that the transgene and maternal genes promoting late flowering, long flowering periods and stem thickening may be preferentially associated in the offspring of weedy mothers. However, although time to first flower is a heritable trait in *B. rapa* [28], our experiment did not confirm the gametic association between the transgene and genes promoting late-flowering in the offspring of hybridized weedy plants. Indeed, given the very small numbers of *Bt*-GFP+ seedlings recovered from the experimental populations, we could not study the association between the transgenic trait and other phenotypic traits in weed plant offspring.

We also found that the weedy plants with the highest probability of hybridization produced fewer seeds, despite producing larger numbers of flowers. The most straightforward interpretation of this result is that fecundity was reduced by hybrid crosses. Controlled crosses between the weedy and transgenic plants used in the experiment (unpublished results) and several previous studies [35], [36] have indeed shown that crops and weed×crop hybrids have lower siring success than do weeds. Therefore, our experiment suggests that maternal weeds that flowered late and for long periods are less fit, because they have a higher probability of hybridizing with GM crop plants or hybrids. This may result in counter-selection against this subset of weed phenotypes, and a shorter earlier flowering period. It is noteworthy that this potential evolution in flowering time does not depend on the presence of the *Bt* transgene in the crop, and may even be counter-balanced by positive selection acting on the transgene if the latter was positively associated with maternal genes promoting late flowering and long flowering periods. Recent experiments indeed indicate that the *Bt* transgene does not induce any fitness costs in hybrids between transgenic *B. napus* and weedy relatives [37], [38]. It may therefore convey a selective advantage under insect herbivore pressure [39].

In conclusion, our analyses show that phenological differences between weedy birdseed rape and transgenic rapeseed are likely to alter the phenotypic structure of weed populations, by promoting interspecific hybridization in only a subset of weedy plants with specific phenotypes and by altering the fitness of hybridizing weeds. Unfortunately, we could not verify the non-random association between the transgenic trait and other phenotypic traits in the offspring of weedy populations because of the very low rate of transgene introgression.

## Materials and Methods

### Experimental design

Nine populations, each composed of 15 *Brassica rapa* plants and 15 of one of three types of transgenic plants (see below) were sown as seeds and then grown from germination until death in a glasshouse at the University of California, Irvine. The nine populations were divided into three blocks, with each transgenic type replicated once per block. Plants were grown in individual Conetainer® (3.8×21 cm) pots filled with a 75/25 mixture of potting soil and sand. Before planting, seeds were vernalized on wet filter paper at 4°C for 5 days. Pots were spaced 7.6 cm apart and were watered every day until 90% stopped producing flowers. An equal amount of 10∶10∶10 NKP liquid fertilizer was applied to each pot on the sowing date.

The three types of transgenic plants were: *Bt*-transgenic *B. napus* crop plants, *Bt-*transgenic *B. napus* × *B. rapa* F_1_ hybrids, and first-generation backcrosses (*B. rapa* ×F_1_ hybrids). Over 20 unique seed and 20 unique pollen parents were used to produce each of the three types. *B. rapa* plants served as seed parents for the F1 and backcross types. *B. napus* were all homozygous for the *Bt-*GFP insertion, whereas the F1 plants were all hemizygous. The backcross generation was expected to consist of an equal mixture of hemizygotes and null homozygotes for the insertion.

*B. rapa* seeds were obtained from over 400 mature plants in a population at Back Bay, near Irvine, California [40]. Transgenic *B. napus* plants were derived from spring rapeseed lines (variety Westar, supplied by Dr. Neal Stewart, University of Tennessee). In addition to the *Btcry1Ac* gene from *Bacillus thuringiensis* (*Bt*) [22], these lines contained a green fluorescent protein (GFP) gene (mGFP5er) under the control of the cauliflower mosaic virus 35S promoter and a nopaline synthase terminator cassette [32], [33]. The fate of the *Bt* transgene could therefore be inferred by exposing the offspring to UV light [32], [34].

Flowering schedules were constructed for each individual plant by recording the time to first flower (i.e., the number of days between sowing and the first observed flower) and the number of opened flowers on every fourth day until the end of the flowering period. The lifetime of a flower is about three days (Weis A., pers. obs.), so this procedure made it possible to estimate the total number of flowers produced by each plant over the flowering period. The length of the flowering period was defined as the number of scoring days on which the plant had opened flowers.

Every fourth day, all open flowers on all plants were hand pollinated in each of the nine experimental populations (there were no natural pollinators in the experimental glasshouse). Each experimental population was composed of 30 plants which were numbered from 1 to 30. On each pollination day, a random sequence of 30 numbers (without repetition) was generated for each population. For a given population, a pollination session consisted of brushing all the flowers of the first plant in the sequence, and then brushing all of the flowers of the next plant. This was continued until the brush from the 30^th^ plant was used to transfer pollen to the first plant. Each plant was brushed up and down several times to deposit the pollen from the previous plant in the sequence and collect the maximum amount of pollen. A given plant was only brushed if it was alive and had one or more open flowers. Otherwise the next plant in the sequence was considered. Each of the nine populations had its own brush, and new brushes were used for each pollination session. This hand-pollination procedure was chosen to approximate the behaviour of a bumble bee in a patch of oilseed rape. Bumblebees tend to visit many plants successively and rarely revisit the plants [41]. They deposit most of the pollen from a source plant on immediate neighbours [42].

We did not keep track of the random sequences of plants generated for each experimental population on each pollination day so we used observed flowering schedules to calculate the expected proportion of pollen received from transgenic plants (*PPR*) for each weedy plant. On each pollination day, the probability of a weedy plant receiving pollen from a transgenic plant was assumed to be proportional to the number of transgenic plants in flower in the experimental population. Over the entire flowering period:
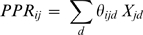
where *PPR_ij_* is the expected proportion of flowers crossed with a transgenic plant for weedy plant *i* from population *j*, *θ_ijd_* is the proportion of flowers open on pollination day *d* for the weedy plant *i* from population *j*, and *Χ_jd_* is the proportion of plants in flower that were of the transgenic type on pollination day *d* in population *j*. The proportion *Χ_jd_* was calculated by excluding the focal plant *i*, since *B. rapa* is known to be largely self-incompatible [43].

In addition to phenological traits, several morphological and reproductive traits were assessed. On the day of first flower, we recorded basal stem diameter and stem height. Dry siliques were collected once the plants had died. The aggregate mass of filled seeds was determined for each plant by separating these seeds from the lighter, aborted seeds, using an air-flow system. We selected five seeds per plant at random and weighed them, to estimate the total number of seeds per plant. We confirmed the accuracy of these measures by counting and weighing all the seeds for 47 plants spanning the range of seed masses.

Finally, for each weedy plant of the nine experimental populations described above, 14 randomly chosen seeds were sown and grown until the day of the first flower. If a mother plant had less than 14 seeds in total, all were sown. Growing conditions were identical to those for the parental generation. Each seedling was scored for fluorescence under high-intensity UV light, at the four-leaf stage. At this stage, the petioles and main nerves of the leaves of transgenic plants displayed fluorescence [34]. This made it possible to determine the proportion of *Bt*-GFP+ seedlings for each mother plant. To investigate the association between the transgenic trait and phenotypic traits in the offspring, time to first flower was recorded for each seedling and, on the day of the first flower, basal stem diameter was measured.

### Statistical analysis

We performed all statistical analyses with SAS/STAT® software [44]. Plants that died during the experiment were excluded from the analysis and the final data set contained 117 weedy plants.

We first investigated how phenological traits affected the chances of interspecific hybridization between *Bt-*trangenic plants and weeds. We used a mixed linear model (SAS, Procedure MIXED), with transgenic type (crop, F1 hybrid or first-generation backcross) as the fixed treatment effect, phenological traits of weeds (time to first flower, flowering duration and total number of flowers) as covariates, and block and treatment×block interaction as random effects. The response variable was the proportion of flowers receiving pollen from *Bt-*transgenic plants (*PPR*). The response variable was log-transformed to increase its normality (Kolmogorov-Smirnov goodness-of-fit; SAS, Procedure UNIVARIATE). If a factor was not significant as a single effect or in interaction with other factors, it was eliminated from the model and the analysis was rerun. We continued until there was no further improvement in residual maximum likelihood.

We then investigated how morphological traits affected the chances of hybridization. Temporal phenotypic clines were assessed by correlating morphological traits of weeds (with time to first flower (Spearman's rank correlation test; SAS, Procedure CORR). A mixed linear approach (SAS, Procedure MIXED) was then used to determine whether the morphological traits changing with time to first flower had a significant effect on *PPR*. As above, transgenic type (crop, F1 hybrid or first-generation backcross) was treated as a fixed treatment effect, morphological traits were covariates and block and treatment×block interaction were treated as random effects.

We used the mixed linear approach (SAS, Procedure MIXED) with block and treatment x block interactions as random effects, to investigate whether the phenological and morphological traits which were found to favour hybridization of weedy mothers were transmitted to their offspring. In this model, transgenic type (crop, F1 hybrid or first-generation backcross) was treated as a fixed effect, the maternal trait as a covariate and the average offspring phenotypic trait as the response variable. The normality of the response variables was checked (Kolmogorov-Smirnov goodness-of-fit; SAS, Procedure UNIVARIATE), and data was transformed as necessary.

Finally we investigated the relationship between opportunities for hybridization and fecundity in weeds. We used the mixed linear approach (SAS, Procedure MIXED) with transgenic type (crop, F1 hybrid or first-generation backcross) as the fixed treatment effect, *PPR* as the covariate and block and treatment×block interaction as random effects. The response variable was the total number of filled seeds. Its normality was checked with a Kolmogorov-Smirnov goodness-of-fit test (SAS, Procedure UNIVARIATE).

We then checked that the mother plants with the highest expected probability of receiving transgenic pollen (*PPR*) also had the highest proportion of *Bt*-GFP+ seedlings. The proportion of *Bt*-GFP+ seedlings did not follow a normal distribution (Kolmogorov-Smirnov goodness-of-fit; SAS, Procedure UNIVARIATE) and could not be transformed. We therefore checked the effects of transgenic type, *PPR* and block separately, in non parametric one-way ANOVAs (SAS, Proc NPAR1WAY, Kruskal-Wallis test). The correlation between *PPR* and the proportion of *Bt*-GFP+ seedlings was assessed using Spearman's rank correlation test (SAS, Proc CORR).
